# Synergistic Manifestations in Cardiac Cysticercosis Complicated by Snake Bite: A Case Report and Literature Review

**DOI:** 10.7759/cureus.58789

**Published:** 2024-04-22

**Authors:** Raviprakash Meshram, Vikas Vaibhav, Yashpal S, Ashok Singh, Shailesh Parate, Gitanjali Khorwal, Kshitiza Sharma, Rahul Sharma

**Affiliations:** 1 Forensic Medicine and Toxicology, All India Institute of Medical Sciences, Rishikesh, Rishikesh, IND; 2 Pathology/Histopathology/Renal Pathology, All India Institute of Medical Sciences, Rishikesh, Rishikesh, IND; 3 Anatomy, All India Institute of Medical Sciences, Rishikesh, Rishikesh, IND; 4 Mental Health Nursing, All India Institute of Medical Sciences, Rishikesh, Rishikesh, IND

**Keywords:** taenia solium, neglected tropical disease, parasitic infections, cysticercosis complications, cardiac cysticercosis

## Abstract

Cysticercosis presents a prevalent issue on a global scale. Nevertheless, disseminated cysticercosis (DCC) is infrequent; even rarer is asymptomatic DCC. Here, we present a unique case of asymptomatic DCC involving the heart in a young male who came to medical attention following a fatal snake bite, ultimately leading to his demise. Despite the widespread dissemination of cysticercosis affecting multiple organs, the individual remained asymptomatic for the condition.

We present a case of a 23-year-old male who was brought to the emergency department with a history of alleged snake bites. The patient was declared dead upon arrival at the All India Institute of Medical Sciences (AIIMS), Rishikesh, India. Autopsy findings revealed multiple significant cardiac abnormalities, including atheromatous changes with calcification in the root of the aorta and aortic valve, along with numerous collateral vessels originating from the left main coronary artery. Additionally, cystic nodules containing cysticercus larvae were identified within the myocardium, suggesting cardiac cysticercosis. The cause of death was determined to be complications related to the snakebite. This case emphasizes the importance of considering multiple potential etiologies in complex clinical presentations, especially in the tropics.

## Introduction

Cysticercus represents the larval stage of the tapeworm *Taenia solium*. In humans, the definitive host, adult tapeworms, inhabit the small intestine, while the larval forms are located in the skeletal muscle of the intermediate host, typically pigs. The development of cysticercosis in humans involves their role as an alternative to pigs in the *T. solium* life cycle, where eggs must mature within the human small intestine as they would in a pig's intestine. The entry of eggs into the human small intestine can occur through autoinfection, ingestion, or inhalation of food or water contaminated with eggs. Subsequently, these cysticercus migrate through the intestinal wall and are transported by the bloodstream to various tissues, including muscles, brain, and subcutaneous tissues, resulting in clinical symptoms. Disseminated cysticercosis (DCC) represents a rare presentation of a prevalent illness [1]. In 2010, the World Health Organization (WHO) designated it as a neglected tropical disease (NTD), while in 2014, the Food and Agriculture Organization of the United Nations (FAO) classified it as a negligible zoonotic disease (NZD) [2].

We present a case of a young male who suffered a snakebite, and upon examination, significant cardiac abnormalities and cysticercosis were discovered, ultimately contributing to his demise.

## Case presentation

A 23-year-old male was brought to the emergency department on 06/08/2022 with a reported history of a snakebite that occurred around 2 PM the same day. Upon examination, two incised wounds were noted on the upper limb, where the nearby individuals attempted to suck out the venom. Despite efforts to save the patient, he was declared dead upon arrival at All India Institute of Medical Sciences (AIIMS), Rishikesh, India, at 07:22 PM on 06/08/2024.

Autopsy findings

The body was of average build, with a red tourniquet present over the left upper arm. The eyes were congested, and their corneas were hazy. Postmortem hypostasis was present over the dependent parts and the back, except the pressure points, and was fixed. Rigor mortis was present all over the body and well-developed.

**Figure 1:**
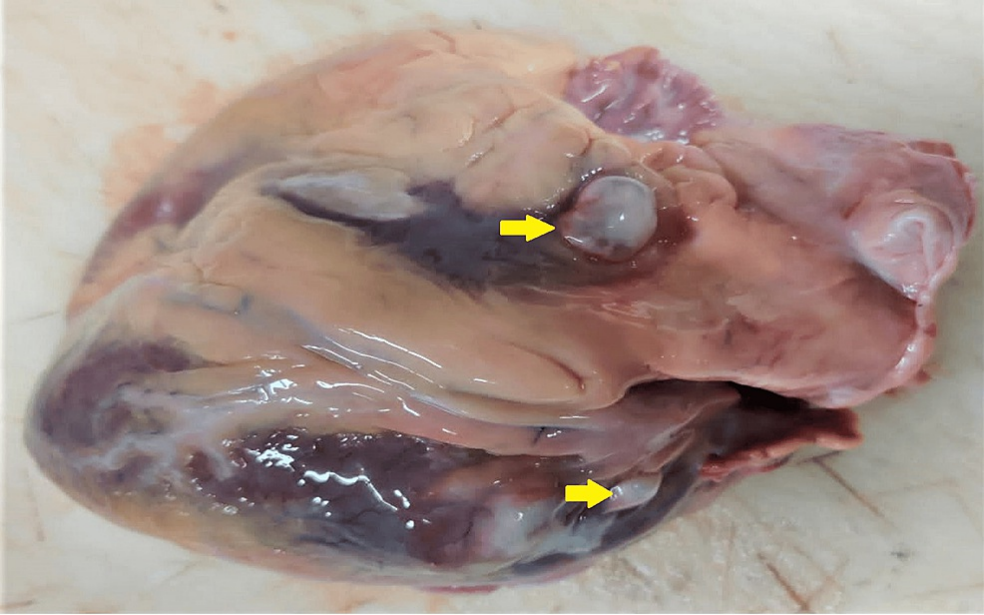
Cystic lesions over the surface of the left atrium

**Figure 2:**
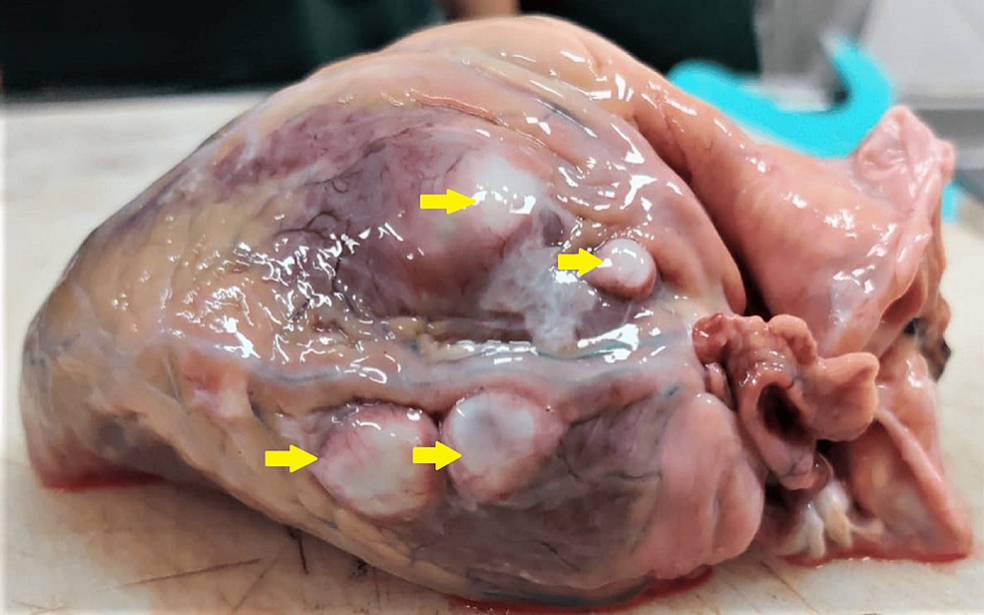
Cystic lesions over the surface of the left ventricle

**Figure 3:**
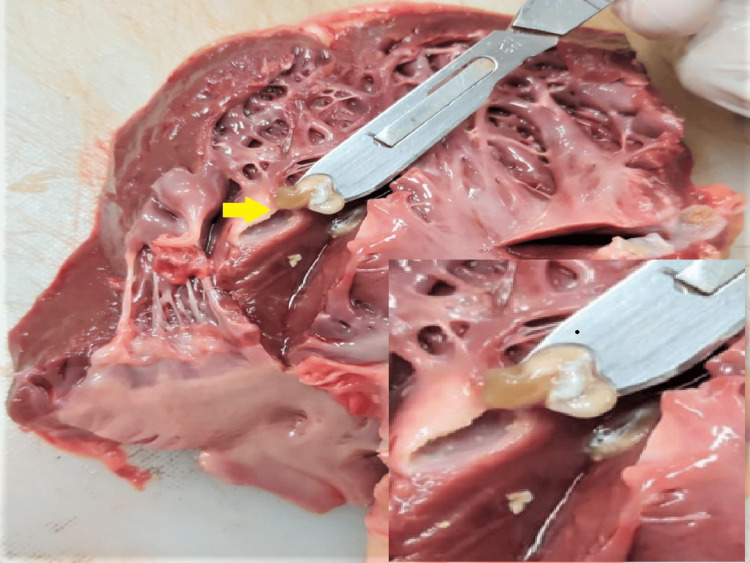
Cystic lesion over the interventricular septum with cysticercus larvae as zoomed in the right corner of this image

**Figure 4:**
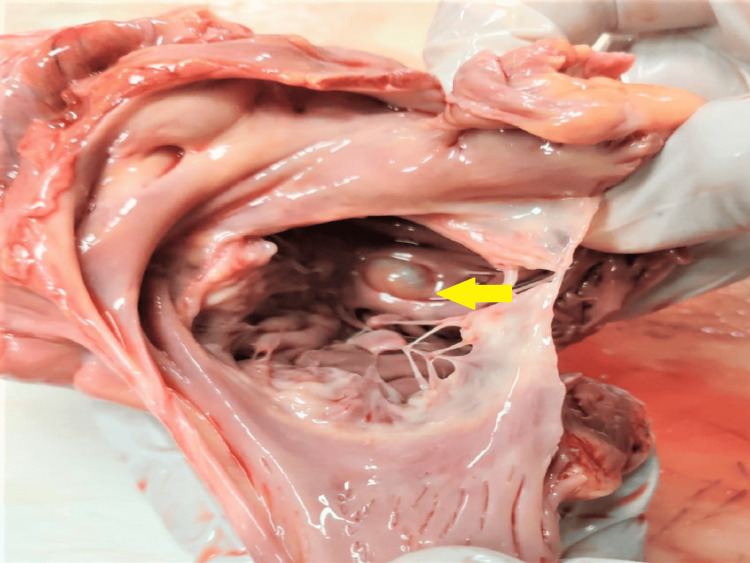
Cystic lesion seen over the root of the aorta

The heart of the deceased was examined during the postmortem examination, revealing a heart weighing 320 grams. The root of the aorta and aortic valve exhibited atheromatous changes with calcification. Multiple collateral vessels were observed, originating from the left main coronary artery and extending along its course. The left main coronary artery itself showed atheromatous changes with a 40% lumen blockage, situated 0.5 cm distal to its origin. Furthermore, numerous cystic nodules with calcified margins containing muddy fluid were discovered on the surface of the left ventricle, the base of the aorta, and the left atrium (Figures 1-3). A similar cystic nodule was identified on the interventricular septum (Figure 4).

**Figure 5:**
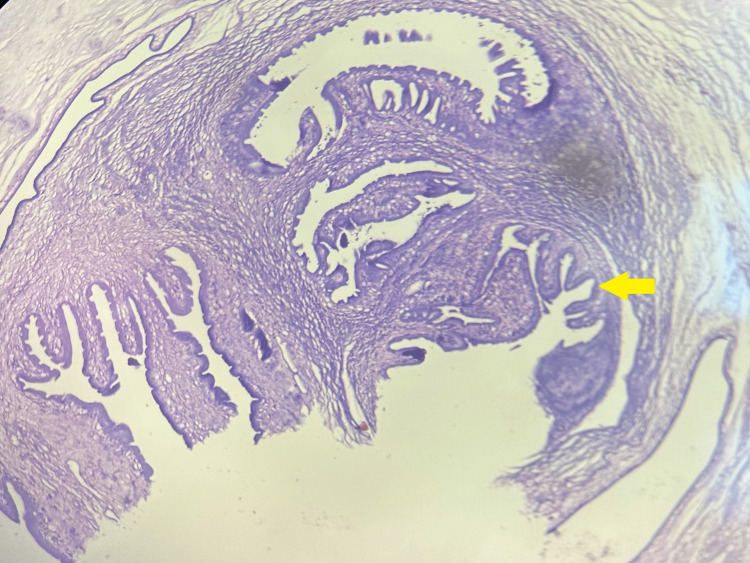
Transverse section showing the hooklets of cysticercus larvae within the myocardium (100x H&E)

**Figure 6:**
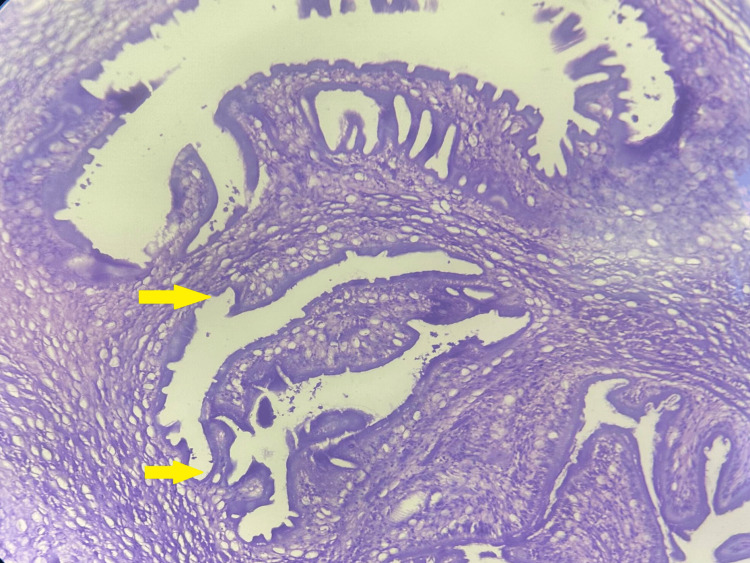
Transverse section showing the hooklets of cysticercus larvae (zoomed in) within the myocardium (100x H&E)

Microscopic examination of heart sections revealed unremarkable cardiac myocytes with focal areas of fibrosis, indicative of an old infarct. Additionally, one of the coronary arteries showed thrombosis with hyalinization of the wall, accompanied by a dense chronic inflammatory cell infiltrate. Intriguingly, during the histopathological examination, a cyst was identified within the myocardium, which was confirmed to contain a cysticercus larva. These features were consistent with cardiac cysticercosis (Figures 5, 6).

## Discussion

This case illustrates the complexity of clinical presentations and the importance of a comprehensive autopsy examination. The patient's history of a snakebite initially seemed to be the apparent cause of death. However, upon further investigation, several significant cardiac abnormalities, including atheromatous changes, coronary artery blockage, and the presence of collateral vessels, were identified. The larval stage of the parasite *T. solium* causes cysticercosis. In regions where the infection is expected, the prevalence of cysticercosis is approximately 1%-2%. The condition primarily affects the central nervous system, subcutaneous tissues, skeletal muscles, abdominal organs, and eyes. Cardiac involvement is considered uncommon in cases of cysticercosis [3]. Autopsy studies have shown that the frequency of cardiac involvement among individuals with DCC can reach up to 27%.

Cysticerci, the larval stage of *T. solium*, can be detected in various heart parts, including the subendocardium, myocardium, and epicardium. They have been found in the left and right ventricular walls, the interventricular septum, the valvular apparatus, and the papillary muscles. These cysticerci typically appear as thin-walled, semi-transparent structures filled with serous fluid. They are oval, measuring up to 30 mm in diameter, and possess a characteristic scolex [3]. As far back as 1912, British Army medical officers stationed in India reported the widespread dissemination of cysticerci throughout the human body [4]. Following those initial reports, subsequent studies should have emphasized this clinical presentation due to its relative rarity [5]. The primary characteristics of DCC encompass intractable epilepsy, dementia, muscle enlargement, and subcutaneous and lingual nodules.

Notably, focal neurological signs or apparent indications of raised intracranial pressure are generally absent, at least until the later stages of the disease [3,6]. The precise impact of immunity on restricting the frequency or widespread occurrence of the disease in humans is not firmly established. However, an autopsy survey conducted in Mexico revealed a connection between immunodeficiency and neurocysticercosis in children [7]. As observed in this case report, the generalized form of cysticercosis may remain asymptomatic, often detected only during autopsies. Nonetheless, it is plausible that the ultimate cardiac dysfunction exhibited by our patient was exacerbated by the extensive cardiopulmonary infestation [8]. Cysticercosis involving the pulmonary and cardiac systems is infrequent. Typically, the diagnosis is confirmed by observing the resolution of lesions following medical treatment with praziquantel or albendazole [9]. Identifying a scolex within a cystic lesion often indicates a diagnosis of cysticercosis [10]. Recognizing DCC clinically and conducting suitable radiological investigations are crucial as this condition requires well-structured therapeutic management. Patients under treatment with active cysts still carry the risk of encountering severe complications [11].

Additionally, the unexpected discovery of cardiac cysticercosis highlights the importance of considering alternative etiologies in cases with atypical findings. Cysticercosis, caused by the larval stage of *T. solium*, is not typically linked with incidents such as snakebites. Nevertheless, its presence in this case emphasizes the significance of a thorough autopsy examination to uncover all potential contributing factors to the patient's demise. A literature review of cases reported worldwide involving the heart is presented in tabulated form (Table 1) [12-40].

**Table 1:** A review of the literature on cardiac cysticercosis ECG: Electrocardiogram; AV: Atrioventricular; CC: Cysticercosis.

References	Case report location	Patient	Presentation	Site of disease
Prabhakar et al. [12], 1990	India	Male	Cyst invading the interventricular septum and papillary muscles, leading to ventricular arrhythmia and branch block	Heart
Jain et al. [13], 2010	Mumbai, India	19-year-old male	Headache and vomiting, seizures, decreased vision, and bilateral proptosis	Heart, brain, extradural spinal space, muscles, lungs, pancreas, and eyes
Gill et al. [14], 2011	Rohtak, India	30-year-old female	One or few cysts over the myocardium	Heart
Vaidya et al. [15], 2013	New Delhi, India	27-year-old male	Multiple subcutaneous nodules all over the patient’s body	Heart, brain, face, orbit, lungs, pancreas, and spleen
Khandpur et al. [16], 2014	New Delhi, India	48-year-old male	Innumerable soft to firm, deep-seated asymptomatic nodular swellings over the trunk and extremities	Heart, skin, central nervous system, skeletal muscles, eyes, and lungs
Dsilva et al. [17], 2017	Mumbai, India	62-year-old male	Episodes of generalized tonic–clonic seizures and multiple subcutaneous nodules over both calves, arms, and nape of the neck	Heart, brain, subcutaneous tissue, liver, and muscles
Sanjay et al. [18], 2017	Rohtak, India	40-year-old male	Unknown	Heart
Littlewood [19], 2022	UK	38-year-old male	Chest pain and ECG changes suggestive of an ST-elevation myocardial infarction	
Bastos et al. [20], 2007	Brazil	39-year-old male	Dyspnea, physical examination showed multiple subcutaneous nodules, which were predominant in the arms and thorax	Multiple ring-like enhancing brain, hypodense nodules present in heart musculature
Nery et al. [21], 2018	Brazil	59-year-old man	Symptoms of typical angina 6 hours in duration	Cystic lesion on the myocardium
Himwaze et al. [22], 2022	Lusaka, Zambia	8 cases, all were male, aged in b/w 28-56 years		Heart, central nervous system, kidney, lung, muscles, and omentum
Lima et al. [23], 2022	Michigan, USA	54-year-old male	Suspected appendicitis; however, a cecal tumor was found	Brain and heart
Spina et al. [24], 2013	Sydney, Australia	24-year-old woman	Having intermittent frontal headaches, high fever, sweats, arthralgias, nausea, vomiting, and weight loss of about 6 kg in one month	Heart and brain
Melo et al. [25], 2005	Brazil	46-year-old female	Exertional dyspnea, palpitations, peripheral edema, ascites, and hepatomegaly	Heart and brain
Thomas et al. [26], 2007	South Africa	42-year-old male	Lightheadedness, bradycardia, and felt dizzy on getting up supine	Heart, brain, muscles, and subcutaneous tissues
Niakara et al. [27], 2002	Burkina Faso, West Africa	37-year-old male	Seizures and bradycardia	Heart, brain, and skin
Sun et al. [28], 1987	China	33-year-old male	Headache, nausea, vomiting, and bradycardia	Heart, skin, and brain
Dediunina et al. [29], 1977	Russia	30-year-old female	Symptomatic bradycardia	Heart and brain
Farina et al. [30], 2023	USA	33-year-old female	Undergoing cardiac surgery	Heart, inferior vena cava, skin, muscle, liver, brain, and larynx
Blandón et al. [31], 2002	Panama	50-year-old male	New York Heart Association stage IV heart failure	Heart and brain
Mauad et al. [32], 1997	Brazil	53-year-old female	Cardiogenic shock and acute myeloid leukemia	Heart, lungs, brain, musculature, and skin
Bhalla et al. [33], 2008	India	35-year-old female	Generalized seizures, proptosis of the right eye, and bilateral calf hypertrophy	Heart, skin, brain, eyes, and musculature
Goldsmid et al. [34], 1966	Cambridge University, Cambridge, UK	Case 1	Died from head trauma. One cyst was found in the autopsy of the myocardium.	Heart
		Case 2	Died from cirrhosis. In the autopsy, 43 cysts were found in the heart.	Heart
Sun et al. [35], 1987	Beijing, China	21-year-old	Patient with complete atrioventricular block, with subcutaneous and cerebral cysticercosis diagnosis.	Heart and brain
Cutrone et al. [36], 1995		Unkown	Man with chest pain and multivessel coronary disease. Ultrafast computed tomography showed multiple cysts in the myocardium of both the right and left ventricles.	Heart
Foyaca-Sibat et al. [37], 2006			Echocardiogram found multiple cysts in papillary muscles and interventricular septum. The patient had bradycardia and II grade AV block.	Brain and heart
Ade et al. [38], 2006	Brazil	26-year-old male	Cerebral and subcutaneous cysticercosis; multiple cysts were found myocardium by echocardiogram.	Brain and heart
Eberly et al. [39], 2008	USA	16-year-old boy	A left ventricular cyst was discovered during a screening echocardiogram.	Heart
Shogan et al. [40], 2009	USA	17-year-old boy	Hypertension with non-enhancing, exophytic, ovoid mural-based fluid density in the left ventricle; biopsy confirmed CC.	Heart

## Conclusions

This case report describes a snakebite complicated by significant cardiac abnormalities and cardiac cysticercosis. The findings underscore the importance of conducting detailed autopsy examinations to evaluate complex clinical cases comprehensively. Recognizing atypical presentations and concurrent pathologies is crucial for gaining a comprehensive understanding of the cause of death, thus informing future medical practices and interventions. Diagnosing disseminated cardiac cysticercosis and conducting required investigations are essential as this condition necessitates strategic therapy. Patients undergoing treatment and still exhibiting active cysts remain vulnerable to severe complications.
